# Sensitive Analysis of Recombinant Human Erythropoietin Glycopeptides by On-Line Phenylboronic Acid Solid-Phase Extraction Capillary Electrophoresis Mass Spectrometry

**DOI:** 10.1021/acs.jproteome.2c00569

**Published:** 2023-02-10

**Authors:** Montserrat Mancera-Arteu, Fernando Benavente, Victoria Sanz-Nebot, Estela Giménez

**Affiliations:** Department of Chemical Engineering and Analytical Chemistry, Institute for Research on Nutrition and Food Safety (INSA·UB), University of Barcelona, Martí i Franquès 1-11, Barcelona 08028, Spain

**Keywords:** capillary electrophoresis, glycopeptides, mass spectrometry, in-line solid-phase extraction, on-line solid-phase extraction, phenylboronic acid

## Abstract

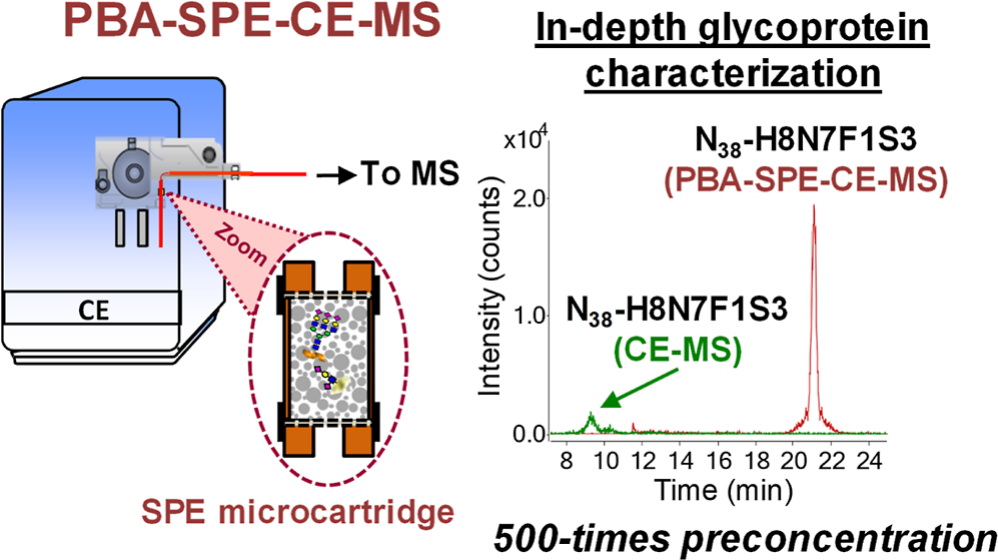

In this study, several chromatographic sorbents: porous graphitic carbon (PGC), aminopropyl hydrophilic interaction (aminopropyl-HILIC), and phenylboronic acid (PBA) were assessed for the analysis of glycopeptides by on-line solid-phase extraction capillary electrophoresis mass spectrometry (SPE-CE-MS). As the PBA sorbent provided the most promising results, a PBA-SPE-CE-MS method was developed for the selective and sensitive preconcentration of glycopeptides from enzymatic digests of glycoproteins. Recombinant human erythropoietin (rhEPO) was selected as the model glycoprotein and subjected to enzymatic digestion with several proteases. The tryptic O_126_ and N_83_ glycopeptides from rhEPO were targeted to optimize the methodology. Under the optimized conditions, intraday precision, linearity, limits of detection (LODs), and microcartridge lifetime were evaluated, obtaining improved results compared to that from a previously reported TiO_2_-SPE-CE-MS method, especially for LODs of N-glycopeptides (up to 500 times lower than by CE-MS and up to 200 times lower than by TiO_2_-SPE-CE-MS). Moreover, rhEPO Glu-C digests were also analyzed by PBA-SPE-CE-MS to better characterize N_24_ and N_38_ glycopeptides. Finally, the established method was used to analyze two rhEPO products (EPOCIM and NeuroEPO plus), demonstrating its applicability in biopharmaceutical analysis. The sensitivity of the proposed PBA-SPE-CE-MS method improves the existing CE-MS methodologies for glycopeptide analysis and shows a great potential in glycoprotein analysis to deeply characterize protein glycosites even at low concentrations of the protein digest.

## 1 Introduction

Glycosylation is one of the most relevant modifications in proteins. Alterations in protein glycosylation have been described in many diseases such as important inflammatory processes and several types of cancer.^1^ On the other hand, the glycosylation pattern of recombinant glycoproteins, which are frequently used as biopharmaceuticals, affects biological activity and pharmacokinetics of the recombinant products, and it can cause an adverse immune response if it differs with respect to the endogenous one.^2^ Recombinant human erythropoietin (rhEPO) is a widely used biopharmaceutical in the treatment of certain forms of anemia. Several rhEPO biosimilars have been commercialized worldwide, reducing the cost of the treatments.^2^ However, it is still necessary to develop novel analytical platforms based on mass spectrometry (MS) not only to improve the quality control of the existing rhEPO biosimilars but also to deeply characterize those products that are under investigation for other clinical applications. This is the case of NeuroEPO plus, a recently developed rhEPO with a low sialic acid content that is currently in phase II–III clinical trials in Parkinson’s and Alzheimer’s diseases.^3,4^ Among the different MS-based strategies to analyze protein glycosylation, the bottom-up analysis of the glycopeptides obtained after enzymatic digestion of the target glycoprotein offers important advantages. Indeed, it provides information not only about the glycan structures but also about the amino acids to which they are attached and hence about the glycosites of the carrier protein.^5^

With regard to the analytical techniques in glycoprotein research, capillary electrophoresis coupled to mass spectrometry (CE-MS) has proved to be a very attractive alternative to liquid chromatography mass spectrometry (LC-MS) for glycopeptide analysis due to its complementary separation mechanism, high separation efficiency, short analysis time, and very low sample and solvent consumption, among others.^6−11^ Moreover, on-line solid-phase extraction capillary electrophoresis mass spectrometry (SPE-CE-MS) has proved to be a very convenient and efficient approach to improve the limits of detection (LODs) of CE-MS. In the most common and simple SPE-CE configuration (*i.e.*, unidirectional and valve-free), a microcartridge containing an affinity sorbent is integrated near the inlet of the separation capillary to clean up and preconcentrate the target analytes from a large volume of the sample, before elution, electrophoretic separation, and detection.^12,13^ Selection of the most appropriate sorbent for optimum performance in SPE-CE-MS is not an easy task. Not only should sorbents show high affinity and selectivity for the target analyte but also their physical properties (*e.g.*, particle shape and size or pore diameter in particulate sorbents) have to be adapted to the reduced dimensions of the microcartridges and separation capillaries and to the fact that the extraction is undertaken on-line with a voltage-driven separation coupled to MS. Until now, only a few sorbents have been used in SPE-CE-MS for the analysis of glycosylated compounds, namely, a weak anion exchange and reversed-phase mixed-mode sorbent for glycans,^14^ an immunoaffinity sorbent for transferrin glycoprotein,^15^ and a titanium dioxide (TiO_2_) sorbent for glycopeptides.^16^ This last sorbent was successfully applied for O-glycopeptides but showed certain limitations for the analysis of N-glycopeptides.

The most commonly used approaches for the off-line purification and enrichment of glycopeptides are lectin affinity, hydrophilic interaction (HILIC), anion exchange, and boronate affinity chromatography-based techniques.^17^ However, with lectins, only a subset of glycopeptide glycoforms can be enriched, and a combination of different lectins is usually required. Otherwise, HILIC sometimes lacks selectivity for certain types of glycopeptides as it is necessary to combine it with anion exchange chromatography to capture a broad range of N- and O-glycopeptides in a single purification step.^17^ In contrast, boronate affinity chromatography can be employed for the selective isolation of glycopeptides containing mannose, galactose, or glucose since boronic acid can form at high pH covalent bonds with the *cis*-diol groups of these saccharides to generate stable cyclic boronate esters. Moreover, interferences retained by noncovalent interactions can be properly washed out before elution of the glycopeptides under acidic conditions that can be compatible with MS detection. Several authors have reported the use of commercially available boronic acid sorbents,^18,19^ or synthesized nanomaterials like metal oxides, metal organic frameworks, and carbon-based and organic polymers functionalized with different boronic acid derivatives to selectively enrich glycopeptides.^19−25^ However, all the proposed methodologies have been implemented off-line, before separation and detection of these analytes of interest.

This study starts with the evaluation of several chromatographic sorbents with the potential for the analysis of glycopeptides by SPE-CE-MS: porous graphitic carbon (PGC), aminopropyl-HILIC, and phenylboronic acid (PBA). As the PBA sorbent provided the most promising results, a PBA-SPE-CE-MS method was developed to selectively retain and enrich glycopeptides from protein digests. The method was optimized and validated for the analysis of O- and N-glycopeptides of the European Pharmacopeia rhEPO reference standard digested with trypsin and Glu-C. Then, results were compared to the ones previously obtained by TiO_2_-SPE-CE-MS to disclose the greater potential of PBA-SPE-CE-MS for the sensitive, reliable, and high-throughput targeted analysis of glycopeptides from protein digests. Finally, the established method was applied to the analysis of EPOCIM and NeuroEPO plus products.

## 2 Experimental Section

### 2.1 Chemicals

All buffers and solutions were prepared with analytical reagent grade chemicals. Acetic acid (HAc, glacial), formic acid (HFor 98–100%), sodium hydroxide, sodium citrate tribasic, and ammonia (25%) were provided by Merck (Darmstadt, Germany). DL-Dithiothreitol (DTT, ≥99%), iodoacetamide (IAA, ≥98%), ammonium acetate (≥99.9%), and ammonium hydrogen carbonate (≥99.9%) were supplied by Sigma-Aldrich (St. Louis, MO, USA). Propan-2-ol was purchased from Scharlab (Barcelona, Spain), while acetonitrile and water were supplied by Sigma-Aldrich (all of them were of LC-MS quality grade). Trypsin and Glu-C (both sequencing grade modified) were provided by Promega (Madison, WI, USA). The ESI low concentration (ESI-L) tuning mix for tuning and calibration of the mass spectrometer was obtained from Agilent Technologies (Waldbronn, Germany).

### 2.2 Recombinant Human Erythropoietin Samples

rhEPO produced in Chinese hamster ovary (CHO) cell lines was provided by the European Pharmacopoeia as a chemical reference substance (CRS-batch 1). Each sample vial contained 100 μg of rhEPO (EPO-CRS; a mixture of epoetin alpha and beta), 0.1 mg of Tween-20, 30 mg of trehalose, 3 mg of arginine, 4.5 mg of NaCl, and 3.5 mg of Na_2_HPO_4_. The content of each vial was dissolved in water to obtain a 1000 mg·L^–1^ solution of rhEPO. Two rhEPOs produced in CHO cell lines were provided by the Center of Molecular Immunology (Havana, Cuba): EPOCIM (batch 1) and NeuroEPO plus (batch 1). EPOCIM vials contained 963 mg·L^–1^ rhEPO and 0.02% (m/v) Tween-20 in citrate buffer at a pH of 6.9. NeuroEPO plus vials contained 1090 mg·L^–1^ rhEPO and 0.02% (m/v) Tween-20 in phosphate buffer at a pH of 6.3. Excipients of low molecular mass were removed from rhEPO samples by centrifugal filtration using Microcon-10 kDa centrifugal filters (Millipore, Molsheim, France) as described in a previous work.^7^ Samples were centrifuged at room temperature in a Mikro 20 centrifuge (Hettich, Tuttligen, Germany). The filter membrane was initially washed with water at 13,000 g for 10 min. Then, the sample was centrifuged, and the residue was washed three times with an appropriate volume of water under the same centrifugal conditions. Finally, the residue was recovered from the upper reservoir by centrifugation upside down into a new vial (3 min at 1000 g), and sufficient water was added to adjust rhEPO concentration to 1000 mg·L^–1^. Aliquots were evaporated to dryness in a Savant SPD-111V SpeedVac concentrator (Thermo-Fisher Scientific, Waltham, MA, USA) and stored at −20 °C until enzymatic digestion.

rhEPO samples were first reduced and alkylated to facilitate digestion. Briefly, an aliquot of 50 μg of dried glycoprotein was dissolved in 50 μL of digestion buffer (50 mM NH_4_HCO_3_, pH 7.9), and 2.5 μL of 0.5 M DTT in digestion buffer was added. The mixture was incubated in a thermoshaker at 56 °C for 30 min. Then, alkylation was carried out by adding 7 μL of 50 mM IAA in digestion buffer and shaking for 30 min at room temperature in the dark. Low molecular mass reagents were removed using Microcon YM-10 centrifugal filters (Millipore) as described above. The final glycoprotein residue was dissolved in digestion buffer to obtain a final concentration of 1000 mg·L^–1^. Aliquots of 50 μL of reduced and alkylated rhEPO solution were digested in an enzyme to a protein ratio of 1:40 (m/m) and incubated at 37 °C for 18 h (trypsin digestion) and then to a protein ratio of 1:20 (m/m) and incubated at 25 °C for 18 h (Glu-C digestion). Digestions were stopped by heating at 100 °C for 10 min, and samples were dried in a SpeedVac before storage at −20 °C until analysis.^7^ Incubations were performed in a TS-100 thermoshaker (Biosan, Riga, Latvian Republic). pH measurements were carried out using a Crison 2002 potentiometer and a Crison electrode 52-03 (Crison instruments, Barcelona, Spain).

### 2.3 CE-MS

CE-MS experiments were performed in a 7100 CE system coupled with an orthogonal G1603 sheath-flow interface to a 6220 oa-TOF LC/MS spectrometer equipped with ChemStation and MassHunter softwares (Agilent Technologies). The sheath liquid [50:50 (v/v) iPrOH/H_2_O with 0.05% (v/v) of HFor] was sonicated for 10 min before being delivered at a flow rate of 3.3 μL·min^–1^ by a KD Scientific 100 series infusion pump (Holliston, MS,USA). The TOF mass spectrometer was operated in the ESI+ mode, and the instrumental parameters were optimized in a previous work^6^ for the analysis of rhEPO O_126_ and N_83_ glycopeptides.

A bare fused-silica capillary of 70 cm total length (*L*_T_) x 75 μm internal diameter (ID) x 360 μm outer diameter (OD) (Polymicro Technologies, Phoenix, AZ, USA) was used in CE-MS. Activation and conditioning procedures were carried out off-line in order to avoid contamination with NaOH of the mass spectrometer. New capillaries were activated by flushing (930 mbar) sequentially for 30 min each with 1 M NaOH, water, and the background electrolyte (BGE, 50 mM HAc and 50 mM HFor, pH 2.2). Capillaries were conditioned every day by flushing with NaOH (5 min), water (7 min), and the BGE (10 min). Samples were reconstituted with the BGE and injected for 15 s at 50 mbar. Electrophoretic separations were performed at 25 °C and 25 kV under normal polarity (cathode in the outlet). Between runs, capillaries were flushed with water (1 min), 1 M HAc (3 min), water (1 min), and the BGE (5 min). Capillaries were stored overnight filled with water. Before CE-MS, all solutions were passed through a 0.22 μm nylon filter (MSI, Westboro, MS, USA).

### 2.4 SPE-CE-MS

A double-frit particle packed fused-silica microcartridge (0.7 cm *L*_T_ × 250 μm ID × 360 μm OD) filled with the SPE sorbent was inserted at 7.5 cm from the inlet of a CE-MS separation capillary as described in our previous studies.^12,13^ PBA (≤40 μm), aminopropyl-HILIC (≤55 μm), and PGC (≤30 μm) from Bond Elut PBA (Agilent Technologies), GlycoWorks HILIC (Waters, Milford, MA, USA), and Hypercarb (Thermo Fisher Scientific, Waltham, MA, USA) SPE cartridges, respectively, were used as sorbents. Before the analyses, the SPE-CE capillaries were checked for abnormal flow restriction flushing water (for aminopropyl-HILIC and PGC sorbents) or 30:69:1 ACN/H_2_O/HFor (v/v/v) (for PBA sorbent) using a syringe and an appropriate connector. Then, capillaries were filled with the BGE, and current stability was checked applying the separation voltage. In the case of PGC-SPE-CE-MS, no electrical current flow was achieved despite using several BGEs and different conditions, as explained later.

#### 2.4.1 Aminopropyl-HILIC-SPE-CE-MS

Under the optimized conditions, the aminopropyl-HILIC sorbent was first conditioned by flushing (930 mbar) with 70% ACN (v/v) for 2 min. Afterward, rhEPO digests were reconstituted in 70% ACN (v/v) to the desired concentration and were loaded by flushing for 10 min (∼60 μL, estimated with the Hagen–Poiseuille equation^26^). A final flush with 70% ACN (v/v) for 2 min removed non-specifically retained molecules. All these initial steps were performed with the nebulizer gas and the ESI capillary voltage switched off to prevent the entrance of contaminants into the mass spectrometer. Then, both were switched on, and the BGE (50 mM HAc and 50 mM HFor, pH 2.2) was pushed at 100 mbar for 30 min, while applying a separation voltage of +25 kV at 25 °C for 30 min. Between consecutive analyses, the capillary was flushed with 70% ACN (v/v) for 2 min to avoid carry-over.

#### 2.4.2 PBA-SPE-CE-MS

Under the optimized conditions, the PBA sorbent was first conditioned by flushing (930 mbar) with 30:69:1 ACN/H_2_O/HFor (v/v/v) (1.5 min) and 20 mM NH_4_Ac pH 10 (1.5 min). Afterward, rhEPO digests were reconstituted in water to the desired concentration and were loaded by flushing for 15 min (∼90 μL^26^). A final flush with the BGE (20 mM NH_4_Ac, pH 6.7) for 3 min eliminated non-specifically retained molecules and equilibrated the capillary before the electrophoretic separation. All these previous steps were performed with the nebulizer gas and the ESI capillary voltage switched off to prevent the entrance of contaminants into the mass spectrometer. Then, both were switched on, and a small volume of the eluent [70:15:15 ACN/H_2_O/HFor (v/v/v)] was injected at 50 mbar for 20 s (∼110 nL,^26^ which corresponds to a capillary length of ∼2.5 cm). Separation was conducted at 25 °C and +25 kV for 30 min. Postconditioning to avoid carry-over was performed by flushing with the eluent (0.5 min) and BGE (3 min).

All quality parameters were calculated from data obtained by measuring the peak area and migration time (*t*_m_) from the extracted ion electropherograms (EIEs) of rhEPO glycopeptide model glycoforms from the rhEPO-trypsin digest, considering a mass accuracy of 20 ppm and multiple *m*/*z* ions for each glycoform (the two most abundant molecular ions per glycoform were at least selected, i.e., protonated ions with charges +2, +3, +4, or +5, and the first four peaks of the isotopic envelope for each molecular ion were considered). Intraday precision (*n* = 3) was evaluated as percent relative standard deviation (% RSD) of peak areas and migration times obtained in consecutive analysis of the rhEPO digest at 1000 mg·L^–1^ for CE-MS (*n* = 3) and at 50 mg·L^–1^ for PBA-SPE-CE-MS (*n* = 3). Linearity ranges were investigated by analyzing rhEPO digests between 25 and 1000 mg·L^–1^ for CE-MS and between 1 and 50 mg·L^–1^ for PBA-SPE-CE-MS. The LODs were estimated by analyzing rhEPO digests at low concentrations and selecting the last concentration experimentally detected (S/N ratios where higher than 3). The lifetime of the microcartridges was evaluated by repeatedly analyzing the rhEPO-trypsin digest at a concentration of 5 mg·L^–1^.

## 3 Results and Discussion

### 3.1 Evaluation of Chromatographic Sorbents

This study starts with the evaluation of several chromatographic sorbents with potential for the analysis of glycopeptides by SPE-CE-MS, with the aim of improving the performance of our previous TiO_2_-SPE-CE-MS methodology.^16^ PGC,^18,27−30^ aminopropyl-HILIC,^31−33^ and PBA^19^ were investigated because they were described in the literature for glycan or glycopeptide analysis using different chromatography-based techniques. rhEPO was chosen as the model glycoprotein because of its broad N- and O- glycosylation microheterogeneity and its interest as a biopharmaceutical. For these screening experiments, rhEPO provided by the European Pharmacopoeia (EPO-CRS) was digested with trypsin (rhEPO-trypsin digest), and three glycoforms of the O_126_ and N_83_ glycosites were selected as model glycopeptides (O_126_-H1N1, O_126_-H1N1S1, and O_126_-H1N1S2 and N_83_-H7N6F1S2, N_83_-H7N6F1S3, and N_83_-H7N6F1S4, respectively). Nomenclature used for glycans corresponds to their composition in terms of number of hexoses (H), N-acetylglucosamines (N), fucoses (F), and sialic acids (S). First, PGC was tested due to its excellent performance in the off-line preconcentration of glycans.^27,30^ However, this sorbent gave unsatisfactory results by SPE-CE-MS as the capillaries with the microcartridge packed with PGC did not allow electrical current flow after applying the separation voltage. This fact impeded the on-line analysis of the retained glycopeptides by SPE-CE-MS, even when using high-conductivity hydro-organic elution plugs, several separation electrolytes, and/or applying pressure (<100 mbar) during separation. From our broad experience in SPE-CE-MS, this maybe related with the electrical properties of PGC particles or the ability to provide an appropriate electroosmotic flow, more than with the promoted backpressure, as particle size was in the most appropriate range for SPE-CE-MS (25–100 μm). Then, the aminopropyl-HILIC sorbent was investigated by analyzing a 10 mg·L^–1^ rhEPO-trypsin digest. The off-line purification protocol recommended by the sorbent manufacturer for the analysis of sialylated glycans by matrix-assisted laser desorption ionization MS was used as starting conditions, namely, 90% (v/v) ACN in the conditioning, loading, and washing solution, 1 mM sodium citrate tribasic as the eluent, and 50 mM HAc and 50 mM HFor, pH 2.2 as the BGE.^34^ However, no glycopeptide glycoforms were detected. After testing several eluents (HFor, HAc, water, and NH_4_Ac at different concentrations and pH values and with several percentages of ACN), we realized that the glycopeptides were eluted while the sorbent was washed with the BGE (50 mM HAc and 50 mM HFor, pH 2.2) and the capillary was filled for the separation. Therefore, we adapted the method conditions to elute with the BGE (see the Experimental Section for details), being able to detect the O_126_ glycopeptide glycoform with one sialic acid (O_126_-H1N1S1). To reduce retention of the peptides from the enzymatic digest, the percentage of ACN was decreased from 90 to 70% (v/v) in the conditioning, loading, and washing solution. Under these conditions, the three most abundant O_126_ glycoforms were detected, as can be observed in Figure S1. However, higher sialylated glycoforms were strongly retained, promoting during separation broader peaks (*e.g.*, O_126_-H1N1S2). In the case of the N_83_ glycopeptide, only the most abundant glycoform was detected (N_83_-H7N6F1S4) and at very low intensity (data not shown in Figure S1), which also made us discard this sorbent for SPE-CE-MS analysis. In contrast to PGC and aminopropyl-HILIC sorbents, the PBA sorbent provided better preliminary results in terms of electrical current flow and glycopeptide extraction, and consequently, it was selected to continue our study.

### 3.2 PBA-SPE-CE-MS Optimization

**Figure 1:**
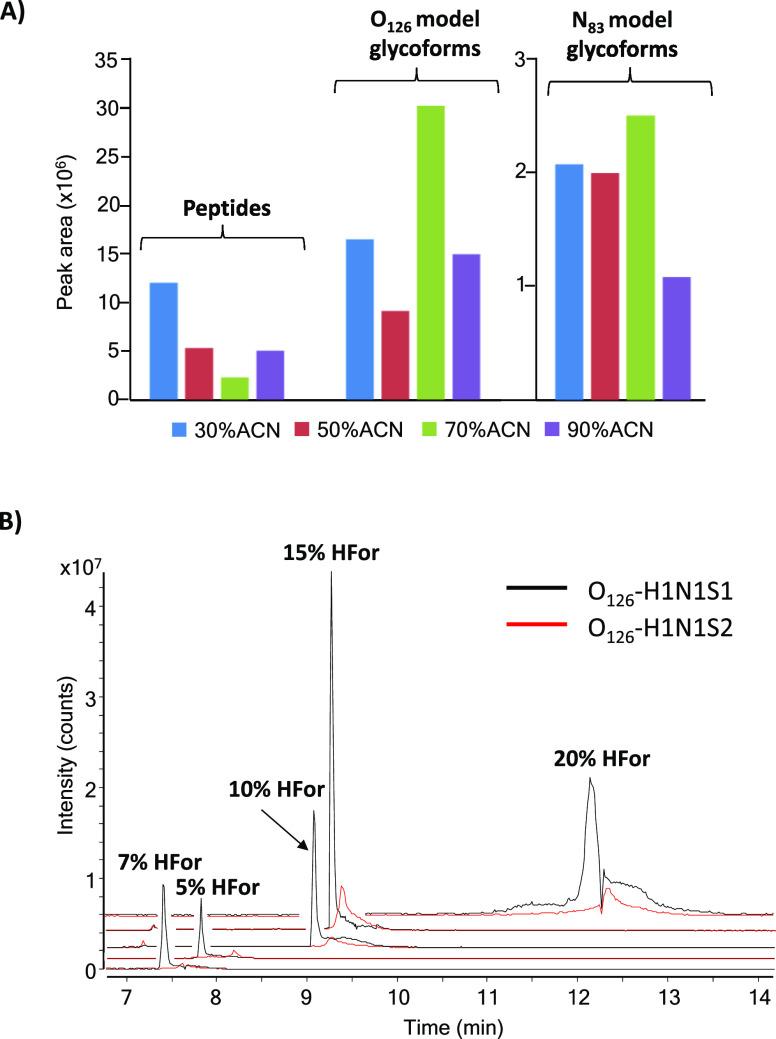
(A) Bar graph showing the effect of the ACN content in the eluent on the peak area of the model O_126_ and N_83_ glycopeptide glycoforms and the total sum of peak areas of peptides of the rhEPO-trypsin digest by PBA-SPE-CE-MS. (B) EIEs of the most abundant model O_126_ glycoforms by PBA-SPE-CE-MS, using several HFor contents in the eluent with 70% (v/v) ACN (concentration of digested EPO-CRS: 10 mg·L^–1^).

Several boronic acid sorbents have been described for the off-line purification and preconcentration of glycoproteins and glycopeptides,^18−25^ but they have never been applied in on-line approaches, including SPE-CE-MS. In our preliminary experiments, PBA microcartridges were first evaluated following the recommendations of the sorbent manufacturer,^35^ with some modifications to avoid the use of non-volatile electrolytes in the conditioning and washing steps. Specifically, the microcartridge was conditioned with 30:69:1 ACN/H_2_O/HFor (v/v/v) and 20 mM NH_4_Ac pH 10. A 10 mg·L^–1^ rhEPO-trypsin digest was loaded for 10 min, and after washing with the BGE (20 mM NH_4_Ac pH 6.7), retained glycopeptides were eluted with 30:60:10 ACN/H_2_O/HFor (v/v/v). The manufacturer recommended for the elution the use of 1% (v/v) HFor,^35^ but in our case, it was necessary to increase the HFor percentage in the elution plug to 10% (v/v) to detect the model glycopeptide glycoforms. These preliminary conditions were optimized, first, by testing several percentages of ACN in the eluent: 30, 50, 70, and 90% (v/v) ACN [with 10% (v/v) HFor]. The bar graph of Figure 1A shows the total peak area of the peptides from the tryptic rhEPO digest and the O_126_ and N_83_ model glycoforms detected by PBA-SPE-CE-MS under different ACN contents. As can be observed, glycopeptide peak areas increased at 70% (v/v) ACN, while peptide peak areas decreased. High percentages of ACN avoided the retention of the glycopeptides by secondary interactions, once they were released from the sorbent by acidification, improving the selectivity of the elution process for detection of the glycopeptides.^35^

As the acidic conditions seemed to be also critical to completely break the covalent bond between the boronate group of the sorbent and the *cis*-diols of the retained glycopeptides, several percentages of HFor in the eluent were also investigated. Figure 1B shows the EIEs of the two most abundant O_126_ model glycoforms for a 10 mg·L^–1^ rhEPO-trypsin digest, with eluents from 5 to 20% (v/v) HFor [with 70% (v/v) ACN]. As can be observed, the highest glycopeptide signal was obtained with 15% (v/v) HFor, with also an adequate separation between glycoforms. Kong *et al.* used this commercial PBA sorbent for the off-line enrichment of glycopeptides by SPE, but results were poorer probably because the elution of the glycopeptides was carried out at lower percentages of HFor [0.5–1% (v/v)] and without ACN.^19^ In our case, we also observed improved results for the rest of O_126_ and N_83_ model glycoforms at 70:15:15 ACN/H_2_O/HFor (v/v/v). Hence, this eluent composition was selected for the analysis of glycopeptides by PBA-SPE-CE-MS.

**Figure 2:**
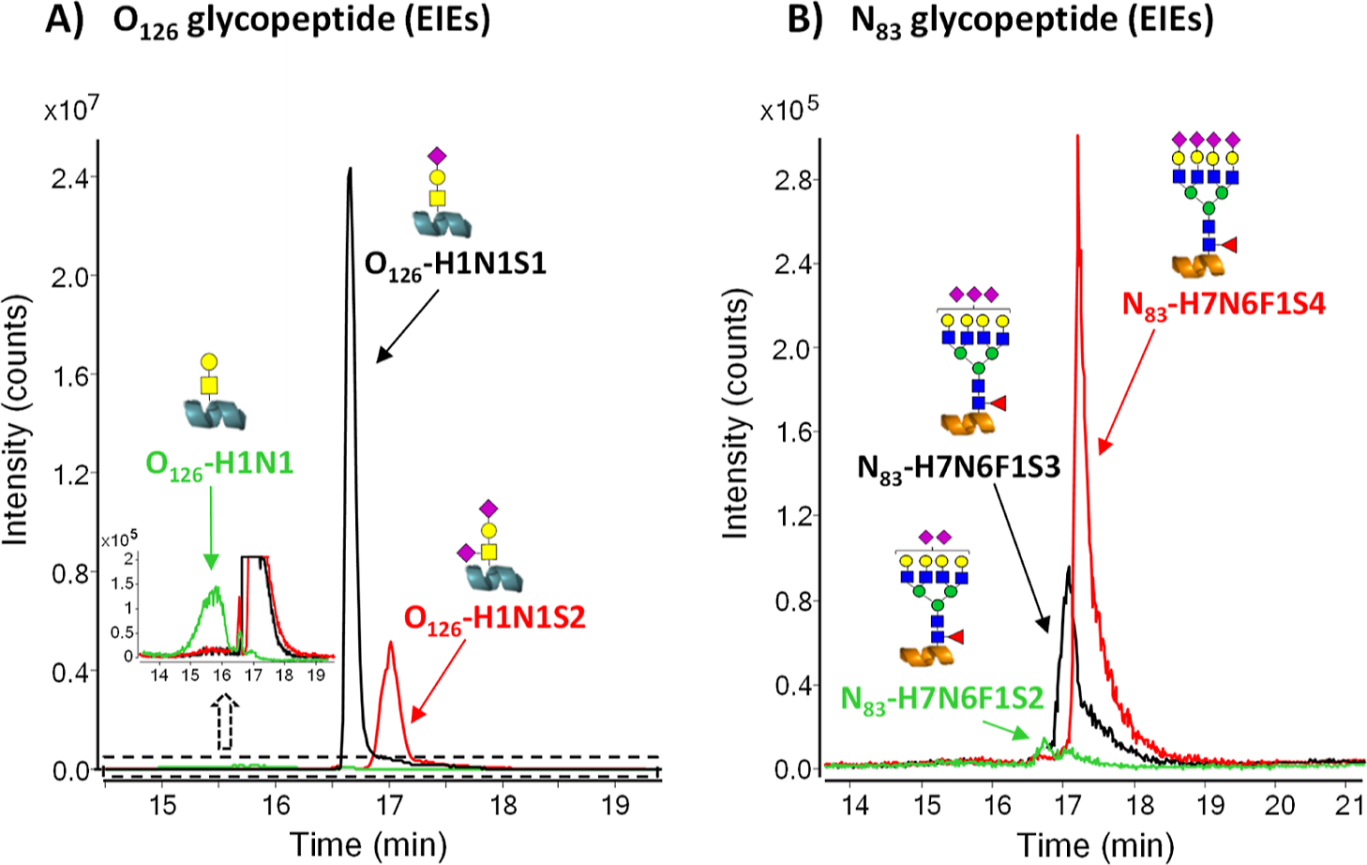
PBA-SPE-CE-MS analysis of a rhEPO-trypsin digest at 50 mg·L^–1^ of digested EPO-CRS under the optimized conditions. EIEs of model (A) O_126_ and (B) N_83_ glycopeptide glycoforms.

Conditioning, washing, and sample loading steps were also studied. As the pK_a_ of the immobilized PBA is ∼9.2, sorbent equilibration with an alkaline solution at a pH of 10–12 is recommended to dissociate boronic acid and obtain the active boronate species before sample loading. With this purpose, after conditioning with 30:69:1 ACN/H_2_O/HFor (v/v/v), 20 mM NH_4_Ac pH 9–12 solutions were tested for sorbent equilibration, and the results obtained for O_126_ and N_83_ glycoforms are depicted in the bar graph of Figure S2. As can be observed, a solution of pH 10 was the one that gave the highest peak areas, especially in the case of N_83_ glycoforms. The composition of the sample loading solution was also investigated, analyzing a 10 mg·L^–1^ rhEPO-trypsin digest reconstituted in water (pH ∼6), 20 mM NH_4_Ac pH 8.5, or 20 mM NH_4_Ac pH 10. The best results were achieved with the digest reconstituted in water, unlike the recommendation of Kong *et al.* (200 mM NH_4_Ac pH 8.5).^19^ With the aim of improving selectivity, a final attempt was made to better remove the non-glycosylated peptides of the digest retained by secondary interactions. To this end, the ionic strength of the washing buffer was increased from 20 to 50 mM NH_4_Ac, and several ACN contents [10, 15, 20, and 30% (v/v)] were also evaluated. Nevertheless, any modification of the washing buffer improved the results, and the BGE (20 mM NH_4_Ac pH 6.7) was selected as the optimized washing solution. Under these conditions, sample loading time was also investigated loading a 5 mg·L^–1^ rhEPO-trypsin digest for 5, 10, 15, and 20 min at 930 mbar (*i.e.*, loading ∼5, 10, 15, and 20 pmol digested EPO-CRS, calculated after estimating the volume with the Hagen–Poiseuille equation^26^). The peak area of O_126_ and N_83_ glycoforms increased progressively from 5 to 15 min and then started decreasing due to analyte breakthrough (see Figure S3A). Therefore, a loading time of 15 min at 930 mbar was selected for the optimized method. By way of an example, Figure 2 shows the EIEs of the model glycoforms of O_126_ and N_83_ glycopeptides obtained by analyzing a 50 mg·L^–1^ rhEPO-trypsin digest under the optimized PBA-SPE-CE-MS method. Compared to CE-MS and the previously established TiO_2_-SPE-CE-MS method,^16^ less separation between glycoforms containing different numbers of sialic acids was achieved, but sensitivity was significantly increased. By way of an example, Figure S4 shows the mass spectra of O_126_ and N_83_ minor and major glycopeptide glycoforms by CE-MS and PBA-SPE-CE-MS. As can be observed, the intensities of the mass spectra substantially increase when analyzing rhEPO digests by PBA-SPE-CE-MS, despite the concentration of digested protein being 20-fold lower than that for CE-MS. In general terms, the most recent nanoC18-LC/MS systems also provide good separation of glycopeptide glycoforms, differing in the number of sialic acids.^36,37^ Nevertheless, PBA-SPE-CE-MS offers shorter analysis times and the instrumentation is simpler, more affordable, and easier to use than the nanoC18-LC/MS system, which requires complex and delicate instrumental setups with valves. Furthermore, as presented before, PBA-SPE-CE-MS efficiently removes the peptides of the digest (Figure 1A). This fact prevents ion suppression effects produced by the peptides, resulting in additional increased glycopeptide sensitivity.

### 3.3 PBA-SPE-CE-MS Method Validation

The PBA-SPE-CE-MS method was validated in terms of linearity, intraday precision, and LODs and compared to CE-MS. Quality parameters were established for the model O_126_ and N_83_ EPO-CRS glycopeptide glycoforms. Table 1 summarizes % RSD values for intraday precision of peak areas and migration times (*n* = 3). The % RSD values ranged from 0.3 to 1.2% for migration times and from 4.9 to 21.0% for peak areas. These values were similar to those obtained by CE-MS. The method was linear between 0.1 and 50 mg·L^–1^ of digested protein for O_126_ and between 0.5 and 50 mg·L^–1^ of digested protein for N_83_ glycoforms (see Table 1).

**Table 1:** Linearity, Intraday Precision of Migration Times and Peak Areas, and LOD for the Analysis of the Model O_126_ and N_83_ EPO-CRS Glycopeptide Glycoforms by CE-MS and PBA-SPE-CE-MS^b^

	linearity		intraday precision^a^				
	regression line (R^2^)	concentration range (mg·L^–1^)	*t*_m_ (min)	% RSD	peak area (×10^6^)	% RSD	LOD (mg·L^–1^)
CE-MS							
O_126_-H1N1	*A* = 277C + 44,998 (0.962)	25–1000	6.8	0.7	0.37	7.1	25
O_126_-H1N1S1	*A* = 20,095C – 1,306,978 (0.997)	25–1000	7.9	1.1	19.53	10.4	5
O_126_-H1N1S2	*A* = 8,256C + 462,586 (0.997)	25–1000	9.5	1.3	8.38	12.7	10
N_83_-H7N6F1S2	*A* = 75C – 4,041 (0.998)	100–1000	8.3	1.5	0.04	5.6	100
N_83_-H7N6F1S3	*A* = 743C – 10,862 (0.988)	25–1000	9.7	1.9	0.32	13.9	25
N_83_-H7N6F1S4	*A* = 4446C – 3902 (0.995)	25–1000	10.2	1.4	1.73	12.1	10
PBA-SPE-CE-MS							
O_126_-H1N1	*A* = 43,380C + 391,381 (0.996)	5.0–50	16.0	1.1	7.38	6.3	1.0
O_126_-H1N1S1	*A* = 6,867,069C + 577,820 (0.999)	0.1–5.0	16.9	1.1	176.91	4.9	0.05
O_126_-H1N1S2	*A* = 954,476C + 1,171,575 (0.977)	0.1–5.0	17.2	1.2	57.70	10.6	0.05
N_83_-H7N6F1S2	*A* = 3,268C – 2,022 (0.970)	0.5–50	17.1	0.3	0.31	21.0	0.5
N_83_-H7N6F1S3	*A* = 27,063C + 50,721 (0.994)	0.5–50	17.3	1.2	2.06	6.4	0.05
N_83_-H7N6F1S4	*A* = 64,483C + 52,000 (0.995)	0.5–50	17.4	1.2	4.76	10.8	0.05

a 1000 mg·L^–1^ (CE-MS) or 50 mg·L^–1^ (PBA-SPE-CE-MS) rhEPO-trypsin digest was analyzed in triplicate on the same day (*n* = 3).

b Nomenclature used for glycans corresponds to their composition in terms of number of hexoses (H), N-acetylglucosamines (N), fucoses (F), and sialic acids (S).

Linearity ranges were narrower than those obtained by CE-MS (25–1000 mg·L^–1^) because when loading higher concentrations, the PBA sorbent was saturated, and the expected proportional increase in the peak areas was not observed. Regarding the LODs obtained by PBA-SPE-CE-MS, they were considerably lower than those obtained by CE-MS, achieving preconcentration factors from 25 to 500 for the model glycoforms. Therefore, the sensitivity enhancement was superior compared to that for TiO_2_-SPE-CE-MS, which only allowed preconcentration factors from 2 to 40 for the same model glycoforms.^16^ Moreover, intraday precision was similar, but the average lifetime of a PBA microcartridge was substantially higher than for a TiO_2_ microcartridge as it could be reused for around 20 consecutive analyses (see Figure S3B). This average lifetime was established by repeatedly analyzing a 5 mg·L^–1^ rhEPO-trypsin digest until the sum of peak areas for the model O_126_ glycoforms in the EIEs decreased more than 30%, compared to the mean value obtained from the fourth to the seventh analyses with the PBA microcartridge under consideration. As can be observed in Figure S3B, the first three injections gave a low signal. This occurred with the PBA sorbent because the microcartridge needed some injections to be completely packed and conditioned. Then, the microcartridge performance decreased between the 16th and 20th injections as the active groups of the small amount of the sorbent became deteriorated due to the large volume of the sample and solutions passed through the microcartridge. It should be noted that the PBA sorbent was manufactured for single use in off-line SPE with conventional cartridges, while here, we demonstrated that it can be reused at the microscale, without substantial changes in performance, for approximately 20 consecutive analyses.

**Figure 3:**
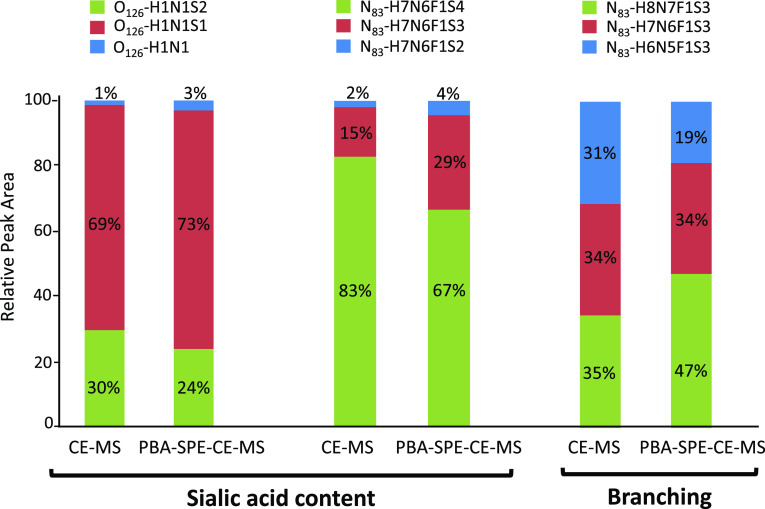
Bar graph showing the influence of the sialic acid content and branching on the relative peak areas of the model O_126_ and N_83_ rhEPO glycopeptide glycoforms by CE-MS and PBA-SPE-CE-MS (Relative peak area was calculated as the peak area of each glycoform divided by the sum of the peak areas of all glycoforms). Concentration of digested EPO-CRS: 1000 and 50 mg·L^–1^ by CE-MS and PBA-SPE-CE-MS, respectively.

As in our previous work,^16^ we evaluated if the sorbent preferentially retained certain glycopeptide glycoforms. With this aim, rhEPO-trypsin digests were analyzed by CE-MS (1000 mg·L^–1^) and PBA-SPE-CE-MS (50 mg·L^–1^). Figure 3 shows the bar graphs for the peak areas of the O_126_ model glycoforms (O_126_-H1N1, O_126_-H1N1S1, and O_126_-H1N1S2) relative to the total sum of their peak areas. The same was represented for the N_83_ model glycoforms (N_83_-H7N6F1S2, N_83_-H7N6F1S3, and N_83_-H7N6F1S4). These representations allowed evaluation of the effect of the sialic acid content in the retention of both glycopeptides. As can be observed, the relative peak areas of O_126_-H1N1S2 and N_83_-H7N6F1S4 by PBA-SPE-CE-MS were slightly lower (24 and 67%, respectively) than by CE-MS (30 and 83%, respectively). To discard the possible desialylation promoted by the high percentage of HFor in the elution plug, we analyzed by CE-MS the same rhEPO tryptic digest reconstituted in water and 15% HFor. No increase of the less sialylated glycoforms were detected upon increasing the acid content (data not shown). This confirmed that the lower relative peak areas detected by PBA-SPE-CE-MS were caused by a certain preference of the PBA sorbent for less sialylated glycoforms, in contrast to what it was observed for the TiO_2_ sorbent.^16^

Retention of glycoforms differing only in the glycan branching was also investigated by representing similar bar graphs for N_83_-H6N5F1S3, N_83_-H7N6F1S3, and N_83_-H8N7F1S3. In this case, the relative peak area of the highly branched glycoform (N_83_-H8N7F1S3) increased compared to that by CE-MS, probably due to its higher *cis*-diol content. Overall, these results demonstrate that, selective chromatographic sorbents used for glycopeptide sample pretreatment provide biased results on the glycoform fingerprint, a fact that most glycoproteomics studies currently overlooked. A similar conclusion may be drawn to other less selective chromatographic sorbents and glycosylated compounds (*e.g.*, glycans). This limitation may be important, for example, when we are interested in obtaining an accurate glycoform glycopeptide map of a certain glycoprotein, but it may be less critical in pathoglycomic studies when comparing between states (*e.g.*, disease *vs* healthy control) to find new glycopeptide glycoforms that could be used as biomarkers.

Finally, to demonstrate the peptide removal efficiency of the optimized method, Figure S5 shows the EIEs of the tryptic peptides of the rhEPO digest and the O_126_ model glycoforms (O_126_-H1N1, O_126_-H1N1S1, and O_126_-H1N1S2) obtained by CE-MS and PBA-SPE-CE-MS. Note that the EIEs of both analyses are shown overlapped at the same intensity scale in order to clearly demonstrate the excellent peptide removal by PBA-SPE-CE-MS. A table with the sequence of the detected tryptic peptides by CE-MS is also depicted in Figure S5. From a total of 13 tryptic peptides detected by CE-MS at 1000 mg·L^–1^ rhEPO, only one peptide (MEVGQQAVEVWQGLALLSEAVLR, highlighted in purple color) was detected by PBA-SPE-CE-MS at 50 mg·L^–1^ rhEPO, while the intensity of O_126_ glycoforms increased considerably due to glycopeptide enrichment. These results confirm the potential of the developed PBA-SPE-CE-MS method for peptide clean-up and glycopeptide preconcentration.

### 3.4 Analysis of rhEPO N-Glycopeptides by PBA-SPE-CE-MS

**Figure 4:**
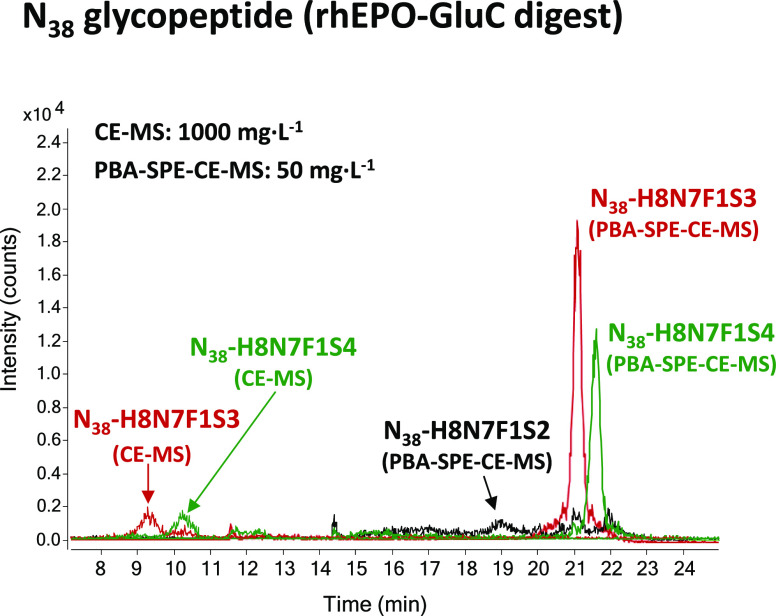
EIEs of a representative set of N_38_ glycopeptide glycoforms analyzed in a rhEPO-GluC digest at 50 and 1000 mg·L^–1^ digested EPO-CRS by PBA-SPE-CE-MS and CE-MS, respectively.

The developed PBA-SPE-CE-MS method was further validated to completely characterize the N-glycosites of rhEPO, including the N_24_ and N_38_ that cannot be properly analyzed digesting with trypsin. For this purpose, EPO-CRS was digested with trypsin (rhEPO-trypsin digest) and Glu-C (rhEPO-GluC digest), and both enzymatic digests were analyzed by PBA-SPE-CE-MS (50 mg·L^–1^) and CE-MS (1000 mg·L^–1^). Table 2 shows the glycoforms detected for each N-glycopeptide (N_24_, N_38_, and N_83_) by PBA-SPE-CE-MS. Note that the N_83_ and N_38_ glycopeptide glycoforms marked with a hashtag were not detected by CE-MS. By way of an example, Figure 4 shows the EIEs of N_38_-H8N7F1 with two, three, and four sialic acids by CE-MS and PBA-SPE-CE-MS. As can be observed, we were able to detect by PBA-SPE-CE-MS, at a 200 times lower concentration of digested protein with Glu-C, the disialylated glycoform (N_38_-H8N7F1S2) and more clearly identify the peaks corresponding to the glycoforms with three and four sialic acids. Therefore, these results suggested that the established sensitive method enabled improving the characterization of rhEPO glycosylation even at low concentrations of digested protein.

**Table 2:** N-Glycopeptide Glycoforms Detected in rhEPO-Trypsin and rhEPO-GluC Digests by PBA-SPE-CE-MS at 50 mg·L^–1^ Digested EPO-CRS^g^

glycopeptide	glycoform	*t*_m_ (min)	*M*_exp_ (Da)^a^	error (ppm)^b^	peak area
N_83_^c^	H6N5F1S2	16.7	5,074.1939	0.4	21,762
	H6N5F1S3	17.1	5,365.2334	10.7	176,262
	H7N6F1S2	16.7	5,439.2890	7.2	27,565
	H7N6F1S3	17.1	5,730.4206	0.5	260,434
	H7N6F1S4	17.2	6,021.4954	3.9	596,425
	H8N7F1S2^#^^f^	16.7	5,804.3882	12.4	36,238
	H8N7F1S3	17.1	6,095.5322	3.8	441,290
	H8N7F1S4	17.2	6,386.6406	1.6	428,422
	H9N8F1S3	17.1	6,460.5866	15.7	35,849
	H9N8F1S4	17.2	6,751.8002	2.5	161,414
	H10N9F1S4^#^^f^	17.2	7,116.9730	8.1	49,638
N_38_^d^	H6N5F1S2^#^^f^	18.7	5,667.3966	5.0	21,164
	H6N5F1S3^#^^f^	21.1	5,958.5026	6.5	77,137
	H7N6F1S2	19.0	6,032.5526	8.6	71,710
	H7N6F1S3	21.1	6,323.6278	5.0	632,147
	H7N6F1S4	21.6	6,614.7286	5.6	451,196
	H8N7F1S2^#^^f^	19.0	6,397.6718	6.1	47,273
	H8N7F1S3	21.1	6,688.7674	5.9	331,339
	H8N7F1S4	21.6	6,979.8754	7.4	240,031
	H9N8F1S3^#^^f^	21.2	7,053.9106	7.1	103,660
	H9N8F1S4	21.6	7,344.9938	5.2	86,199
N_24_^e^	H5N4F1S2	22.0	3,415.2939	5.6	135,972
	H6N5F1S2	22.1	3,780.4218	4.0	40,216
	H6N5F1S3	22.2	4,071.5529	12.4	116,507
	H7N6F1S2	22.1	4,145.4942	10.8	35,054
	H7N6F1S3	22.1	4,436.5968	8.5	90,332
	H7N6F1S4	22.2	4,727.7810	10.8	70,228
	H8N7F1S3	22.0	4,801.8540	18.2	34,931
	H8N7F1S4	22.1	5,092.9875	24.6	14,402

a *M*_exp_ monoisotopic molecular mass.

b Error was calculated in parts per million as follows: (|*M*_exp_ – *M*_theo_|/*M*_theo_) × 10^6^ (exp = experimental and theo = theoretical).

c N_83_ glycopeptide detected in the rhEPO-trypsin digest (N_83_ (77–97)).

d N_38_ glycopeptide detected in the rhEPO-GluC digest (N_38_ (32–55)).

e N_24_ glycopeptide detected in the rhEPO-GluC digest (N_24_ (22–31)).

f # Indicates glycoform not detected by CE-MS at 1000 mg·L^–1^ digested protein.

g Nomenclature used for glycans corresponds to their composition in terms of the number of hexoses (H), N-acetylglucosamines (N), fucoses (F), and sialic acids (S).

### 3.5 Application to rhEPO Biosimilars

**Figure 5:**
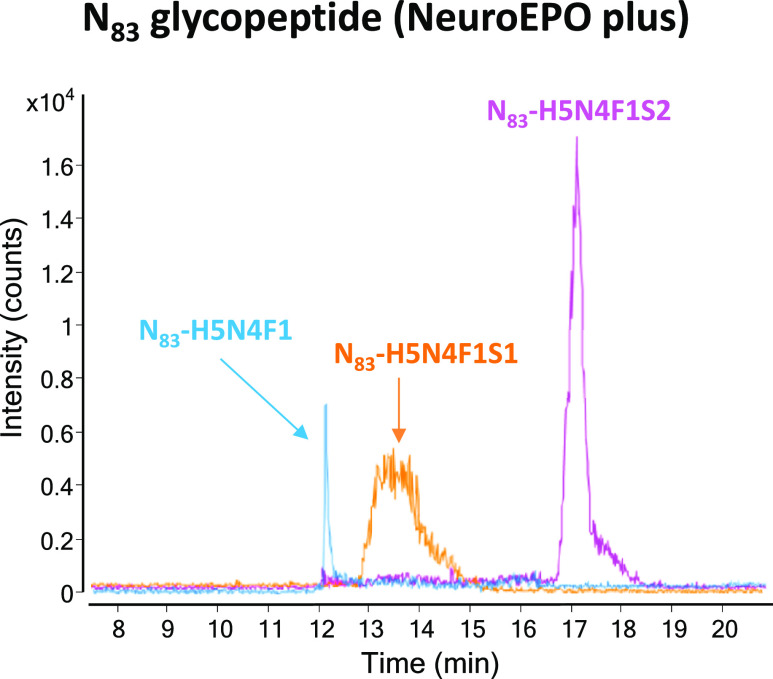
EIEs of N_83_-H5N4F1 sialoforms of NeuroEPO plus detected by PBA-SPE-CE-MS in a rhEPO-trypsin digest at 50 mg·L^–1^ digested NeuroEPO plus.

Finally, the PBA-SPE-CE-MS method was applied to the analysis of two rhEPO products. EPOCIM is a biosimilar commercialized for the treatment of anemias, and its production may result in a slightly different glycosylation pattern from that of EPO-CRS. NeuroEPO plus is a basic rhEPO under investigation for the treatment of neurodegenerative diseases.^4^Table 3 shows the glycoforms of the O_126_ and N_83_ glycopeptides of EPOCIM and NeuroEPO plus detected by PBA-SPE-CE-MS at 50 mg·L^–1^ digested protein. NeuroEPO plus showed a superior amount of less sialylated glycoforms in both O_126_ and N_83_ glycopeptides, while the glycoform composition of EPOCIM was similar to that of EPO-CRS (compare Tables 2 and 3 for N_83_ glycopeptide). This similarity was also found for the less abundant glycoforms with N-glycolylneuraminic acid (NeuGc, S*), which are characteristic of CHO-derived glycoproteins^38^ (*e.g.*, NeuGc represented ∼2% of the O_126_ mono-sialylated glycoforms, H1N1S1 and H1N1S1*, Table 3). If we focus on the great differences found for the N_83_ glycopeptide in NeuroEPO plus with regard to EPOCIM and EPO-CRS (see Tables 2 and 3), they were related not only to the proportion of the detected glycoforms with lower sialic acid content but also to their type and microheterogeneity in terms of branching [*e.g.*, bi-antennary glycoforms (H5N4F1) were exclusively detected]. By way of an example, Figure 5 shows the EIEs of the N_83_-H5N4F1 sialoforms of NeuroEPO plus detected by PBA-SPE-CE-MS. The glycoform composition of this novel rhEPO product will be useful, in the near future, to understand why NeuroEPO plus shows a higher neuroprotective effect than conventional rhEPO without erythropoiesis stimulation. Overall, the obtained results demonstrated the applicability of the established method in biopharmaceutical analysis, to deeply characterize the glycoform profile of biosimilars or products under development, since even a less abundant glycoform can cause a different therapeutical effect or an adverse immunogenic response.

**Table 3:** O_126_ and N_83_ Glycopeptide Glycoforms Detected in EPOCIM and NeuroEPO Plus by PBA-SPE-CE-MS at 50 mg·L^–1^ Digested Protein^f^

		EPOCIM			NeuroEPO plus		
glycopeptide	glycoform	*M*_exp_ (Da)^a^	error (ppm)^b^	peak area	*M*_exp_ (Da)^a^	error (ppm)^b^	peak area
O_126_^c^	N1	1,667.8343	0.7	232,916	1,667.8387	1.9	2,765,528
	H1N1	1,829.8851	1.7	521,938	1,829.8917	1.9	10,493,567
	H1N1S1	2,120.9815	1.0	13,789,432	2,120.9923	4.1	55,116,793
	H1N1S1*^e^	2,136.9749	1.8	360,691	2,136.9827	1.9	1,561,912
	H1N1S2	2,412.0751	1.7	2,850,915	2,412.0851	2.5	13,007,993
	H1N1S1S1*^e^	2,428.0669	3.0	365,883	2,428.0759	0.7	1,168,973
N_83_^d^	H5N4F1				4,126.8803	2.3	41,841
	H5N4F1S1				4,417.9811	3.3	491,132
	H5N4F1S2				4,709.0741	2.5	336,791
	H6N5F1				4,492.1669	36.5	40,073
	H6N5F1S1				4,783.1063	1.6	475,195
	H6N5F1S2	5,074.0454	29.3	46,433	5,074.2044	2.0	513,429
	H6N5F1S3	5,365.2898	0.05	190,445	5,365.2743	2.9	532,779
	H7N6F1				4,857.1823	9.7	38,258
	H7N6F1S1				5,148.2321	0.2	614,151
	H7N6F1S2	5,439.3826	10.4	142,439	5,439.3862	11.0	611,485
	H7N6F1S3	5,730.4418	3.6	384,631	5,730.4226	0.3	618,951
	H7N6F1S4	6,021.5082	1.4	940,745	6,021.5386	3.7	82,126
	H8N7F1S1				5,513.3502	2.2	495,746
	H8N7F1S2	5,804.4830	4.2	169,183	5,804.4239	5.9	605,615
	H8N7F1S3	6,095.5314	3.6	581,080	6,095.6038	8.3	454,383
	H8N7F1S4	6,386.6458	0.4	536,938	6,386.5466	16.0	80,271
	H9N8F1S1				5,878.5686	12.6	232,299
	H9N8F1S2				6,169.6418	8.4	135,119
	H9N8F1S3	6,460.6994	2.2	57,137	6,460.7114	4.0	142,314
	H9N8F1S4	6,751.8190	5.6	89,273	6,751.6390	21.0	43,823

a *M*_exp_ monoisotopic molecular mass.

b Error was calculated in parts per million asfollows: (|*M*_exp_ – *M*_theo_|/*M*_theo_) × 10^6^ (exp = experimental and theo = theoretical).

c O_126_ glycopeptide detected in the rhEPO-trypsin digest [O_126_ (117–131)].

d N_83_ glycopeptide detected in the rhEPO-trypsin digest [N_83_ (77–97)].

e * Indicates one sialic acid is N-glycolylneuraminic acid instead of N-acetylneuraminic acid.

f Nomenclature used for glycans corresponds to their composition in terms of the number of hexoses (H), N-acetylglucosamines (N), fucoses (F), and sialic acids (S).

## 4 Conclusions

We demonstrated that certain chromatographic sorbents widely described for the off-line purification and preconcentration of glycans and glycopeptides have limitations for the on-line analysis of glycopeptides by SPE-CE-MS. PGC showed electrical current flow issues, and aminopropyl-HILIC was difficult to make compatible with an adequate BGE and rapid elution for appropriate electrophoretic separations. In contrast, PBA provided excellent performance to compete with TiO_2_ for the analysis of glycopeptides by SPE-CE-MS. A PBA-SPE-CE-MS method was developed to selectively retain and enrich glycopeptides from rhEPO digests. Under the optimized conditions, linearity and intraday precision, in terms of migration times and peak areas, were adequate and the microcartridge lifetime was longer than by TiO_2_-SPE-CE-MS. PBA-SPE-CE-MS provided lower LODs especially for N-glycopeptides (up to 500 and 200 times lower than by CE-MS and TiO_2_-SPE-CE-MS, respectively). Although the PBA sorbent showed certain preference for some glycopeptide glycoforms, as also happened with the TiO_2_ sorbent, the increased sensitivity of the proposed PBA-SPE-CE-MS method improves the existing CE-MS methodologies for glycopeptide analysis. Moreover, its robustness and excellent performance in analyzing O- and N-glycopeptides at a low concentration of digested protein point to its great potential in biopharmaceutical analysis to deeply characterize protein glycosites, paving the way to analyze glycoprotein biomarkers in biological samples.

## Supporting Information Available

Aminopropyl-HILIC-SPE-CE-MS results; PBA-SPE-CE-MS results using conditioning solutions of 20 mM NH4Ac at different pH values, sample loading time and evaluation of the microcartridge lifetime for consecutive analysis, mass spectra of glycopeptides by CE-MS and PBA-SPE-CE-MS, and peptide and glycopeptide detection by CE-MS and PBA-SPE-CE-MS The mass spectrometry data have been deposited to the ProteomeXchange Consortium *via* the PRIDE partner repository with the dataset identifier PXD039282 (PDF)

## Notes

Data are available via ProteomeXchange with identifier PXD039282.
