# Supplementary material for: Sensitive Analysis of Recombinant Human Erythropoietin Glycopeptides by On-Line Phenylboronic Acid Solid-Phase Extraction Capillary Electrophoresis Mass Spectrometry
Source: J Proteome Res. 2023 Feb 10;22(3):826–36. doi: 10.1021/acs.jproteome.2c00569 (PMC9990126; doi:10.1021/acs.jproteome.2c00569)
Supplement: Supplementary file 1 — pr2c00569_si_001.pdf [file pr2c00569_si_001.pdf]

Supporting Information

SENSITIVE ANALYSIS OF RECOMBINANT HUMAN ERYTHROPOIETIN GLYCOPEPTIDES BY ON-LINE PHENYLBORONIC ACID SOLID-PHASE EXTRACTION CAPILLARY ELECTROPHORESIS-MASS SPECTROMETRY

Montserrat Mancera-Arteu[1], Fernando Benavente[1], Victoria Sanz-Nebot[1], Estela Giménez[1]*

[1] Department of Chemical Engineering and Analytical Chemistry, Institute for Research on Nutrition and Food Safety (INSA·UB), University of Barcelona, Martí i Franquès 1-11, 08028 Barcelona, Spain

*Corresponding author: estelagimenez@ub.edu (E. Giménez, PhD)

Keywords: Capillary electrophoresis; glycopeptides; mass spectrometry; in-line solid-phase extraction; on-line solid-phase extraction; phenylboronic acid

Table of contents

Figure S1: Aminopropyl-HILIC-SPE-CE-MS results S-3

Figure S2: PBA-SPE-CE-MS results using conditioning solutions of 20 mM NH_4Ac at different pH values S-4

Figure S3: (A) Sample loading time and (B) Evaluation of the microcartridge lifetime for consecutive analysis. S-5

Figure S4: Mass spectra of glycopeptides by CE-MS and PBA-SPE-CE-MS S-6

Figure S5: Peptide and glycopeptide detection by CE-MS and PBA-SPE-CE-MS S-7

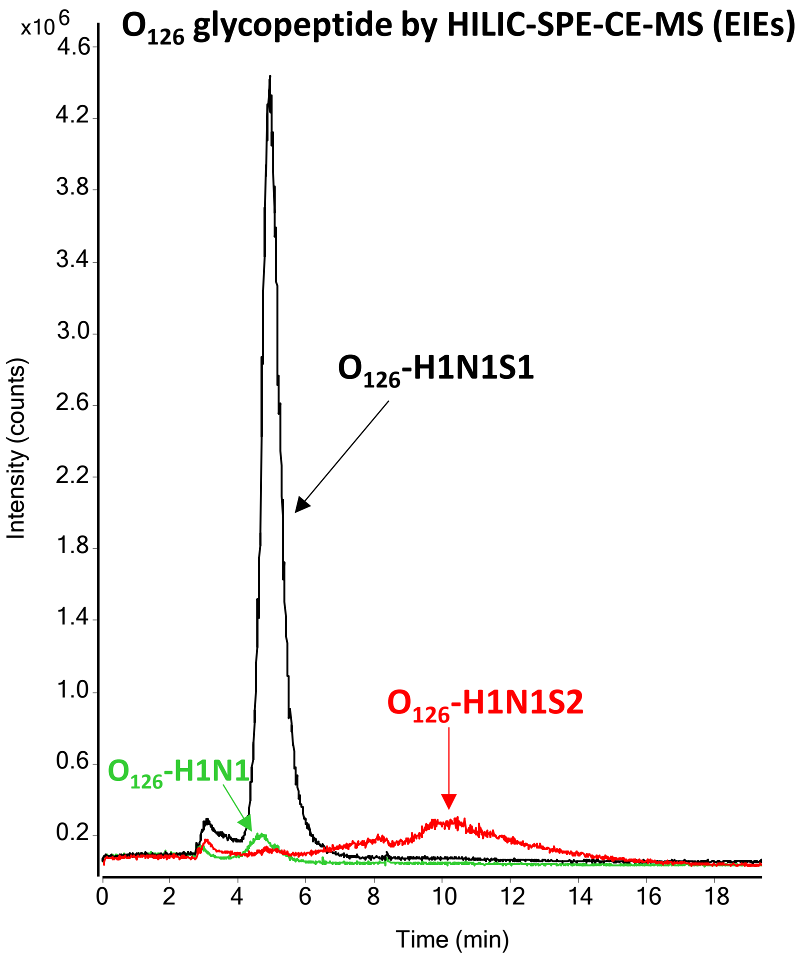

Figure S1: Extracted ion electropherograms (EIEs) of model O_{126} glycopeptide glycoforms analyzed in a rhEPO-trypsin digest at 10 mg·L^{-1} of digested EPO-CRS by aminopropyl-HILIC-SPE-CE-MS.

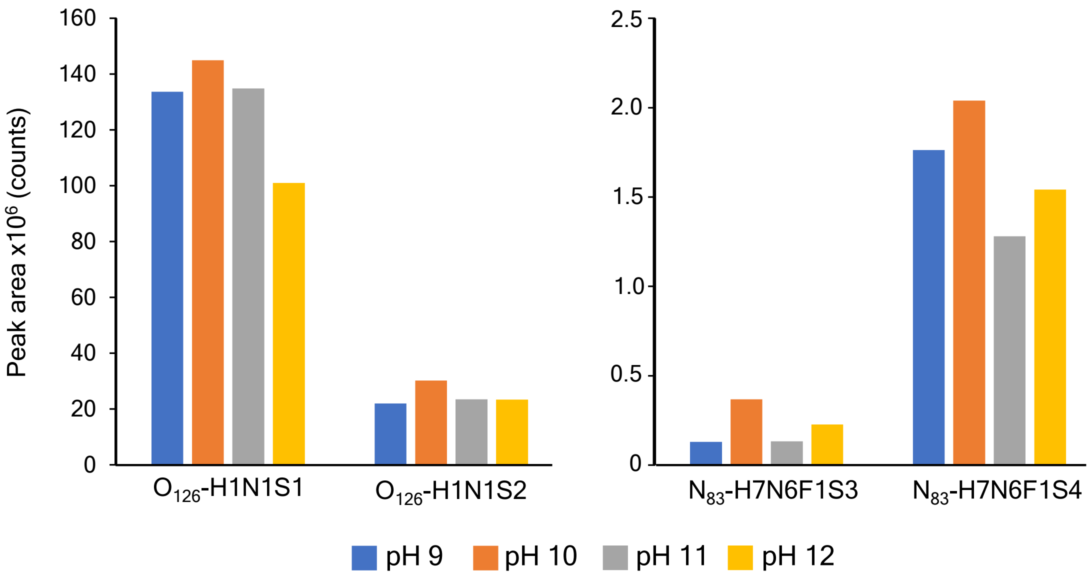

Figure S2: Bar graph showing the peak areas of the most abundant model O_{126} and N_{83} glycopeptide glycoforms by PBA-SPE-CE-MS using conditioning solutions of 20 mM NH_4Ac at different pH values (analysis of a rhEPO-trypsin digest at 10 mg·L^{-1} of digested EPO-CRS).

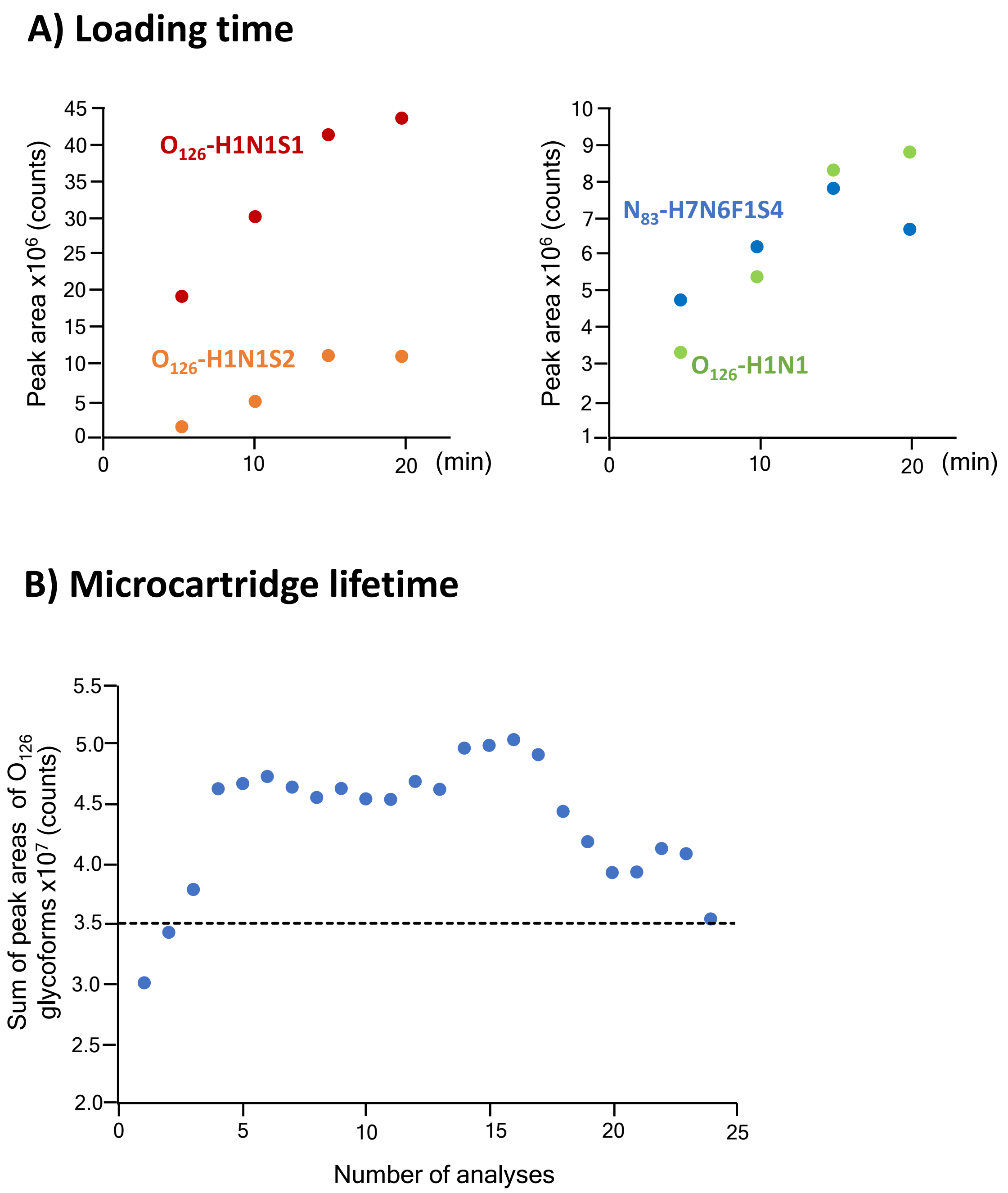

Figure S3: Analysis of a rhEPO-trypsin digest at 5 mg·L^{-1} of digested EPO-CRS by PBA-SPE-CE-MS. (A) Plot of peak areas of the three model O_{126} glycopeptide glycoforms and N_{83}-H7N6F1S4 *versus* loading time at 930 mbar. (B) Evaluation of the microcartridge lifetime for consecutive analysis. (The microcartridge was discarded when the sum of peak areas of model O_{126} glycoforms decreased more than 30% compared to the mean value obtained from the 4th to the 7th analyses).

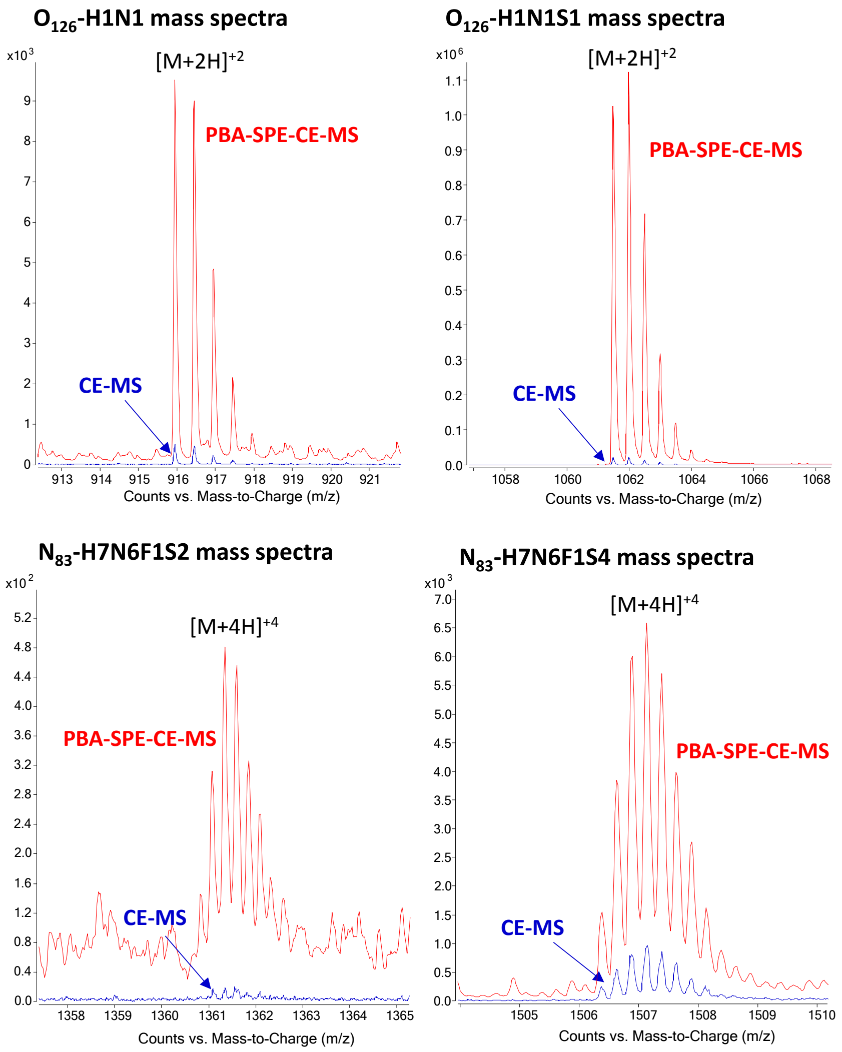

Figure S4: Mass spectra of minor and major glycoforms of O_{126} and N_{83} glycopeptides in a rhEPO-trypsin digest analyzed by CE-MS and PBA-SPE-CE-MS at 1,000 and 50 mg·L^{-1} of digested EPO-CRS, respectively.

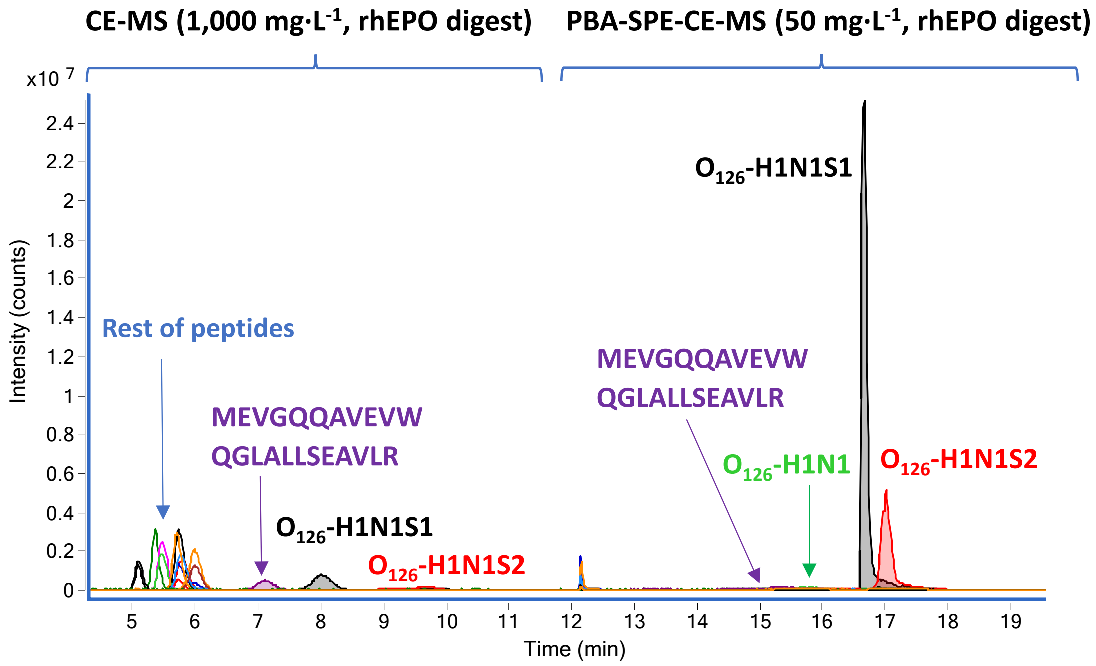

Peptides detected by CE-MS (rhEPO–trypsin digest)	Mass
APPR	439.2543
LICDSR-(Cys-IAA)	762.3694
VLER	515.3067
YLLEAK	735.4167
VNFYAWK	926.465
MEVGQQAVEVWQGLALLSEAVLR	2525.3311
AVSGLR	601.3547
SLTTLLR	802.4912
ALGAQK	586.3438
TITADTFR	923.4712
LFR	434.2641
VYSNFLR	897.4708
LYTGEACR-(Cys-IAA)	968.4385

Figure S5: CE-MS and PBA-SPE-CE-MS analysis of a rhEPO-trypsin digest at 1,000 and 50 mg·L^{-1} of digested EPO-CRS, respectively, under the optimized conditions. EIEs of rhEPO tryptic peptides and O_{126} glycopeptide model glycoforms, and table with the peptide sequence of the rhEPO tryptic peptides detected by CE-MS. The peptide highlighted in purple was the only one also detected by PBA-SPE-CE-MS.
